# Paediatric palliative care in cancer

**DOI:** 10.3332/ecancer.2024.1823

**Published:** 2024-12-12

**Authors:** Julia Downing, Alexandra Daniels, Michael J McNeil, Mariam Ndagire, Gayatri Palat, Rime S Rassam, Justin N Baker

**Affiliations:** 1 International Children’s Palliative Care Network (ICPCN), BS1 2NT Bristol, UK; 2 International Children’s Palliative Care Network (ICPCN), Durban 3624, South Africa; 3 Makerere/Mulago Palliative Care Unit (MMPCU), Kampala, Uganda; 4 St. Jude Global Palliative Care Program, St. Jude Children’s Research Hospital, Memphis, TN 38105, USA; 5 Uganda Cancer Institute, Kampala, Uganda; 6 MNJ Institute of Oncology and Regional Cancer Centre, Hyderabad 500004, Andhra Pradesh, India; 7 Two Worlds Cancer Collaboration, North Vancouver, BC V7H 2Y8, Canada; 8 St. Jude Global Nursing, St. Jude Children’s Research Hospital, Memphis, TN 38105, USA; 9 Division of Quality of Life and Pediatric Palliative Care, Lucille Packard/Stanford University, Palo Alto, CA 94305, USA

**Keywords:** palliative care, paediatrics, cancer, education, oncology, research, medicines, service provision, Ghana, Lebanon, USA, India, Uganda

## Abstract

More than 21 million children globally need access to palliative care (PC) – including children with cancer. Providing Paediatric Palliative Care (PPC) for children with cancer is an ethical imperative with pain relief being recognised as a human right and an important public health consideration, with PPC being essential for reducing suffering in children and families. PPC addresses children’s symptoms and aims to provide comfort even if a cure is not possible. PC for children with cancer is about ensuring that the child and family have the best possible quality of life starting at diagnosis and throughout the disease trajectory regardless of cancer treatment outcomes. Many principles of PPC for children with cancer are similar to those for children with other serious health conditions. These include the following: promotion of quality of life; provision of PC care across the continuum of care (from diagnosis through to bereavement); pain and symptom management; emotional support; social care; spiritual care; good communication with children and family; advance care planning; end-of-life care; and bereavement care. PPC should be provided across the range of care settings, wherever the child and their family need care, by an inter-disciplinary team providing support to the child, their families (including siblings) and other significant others, and consider the financial impact of having a child with cancer. It should not be a last resort, but an essential component of care. In this paper, we provide a brief overview of the integration of PPC into paediatric cancer care through the review of challenges in providing PPC in paediatric oncology, global examples of clinical provision of PPC in paediatric cancer care, a review of global research priorities in this area and examples of global education programmes aimed at improving PPC in paediatric cancer care.

## Introduction

More than 21 million children globally need access to palliative care (PC) [1] including children with cancer. More than 1,000 children (0–19 years) are diagnosed with cancer daily [2] and an estimated 400,000 children globally each year [3, 4], with approximately 90% living in low-and middle-income countries (LMICs) [5]. Cancer is curable in most children, more so when good diagnostics, therapeutics and supportive care services are available. Whilst more than 80% of children with cancer in high-income countries (HICs) survive, this is as low as 15%–45% in children in LMICs demonstrating great disparity and inequity globally [3, 6, 7]. Most common childhood cancers differ by region, with a higher incidence of lymphomas, retinoblastomas and renal tumours in African regions [8]. The consequences of a higher burden of cancer are avoidable deaths, social and economic hardship. When providing PC to children with cancer we ‘cloak’ their symptoms and aim to provide comfort even if cure is not possible thus ensuring that the child and family have the best possible quality of life regardless of disease prognosis. Many principles of paediatric palliative care (PPC) for children with cancer are similar to those for children with other conditions whilst recognising the specific impact of cancer on the child and their families.

## PC in paediatric cancer care

The impact of cancer on children and their families is devastating both in HICs and LMIC, in terms of short and long-term psychosocial, emotional, society and economic consequences secondary to cancer and its treatment. This is compounded in LMICs where an estimated 80% of children present with advanced disease [9] resulting in increased suffering and reduced quality of life with limited access to treatment, medicines, services and trained personnel, and where treatment is available it is often unaffordable or of poor quality [7]. Thus, providing PPC for children with cancer is an ethical imperative, significant and important public health consideration, with pain management being recognised as a human right [10], and essential for both preventing and reducing suffering in children and families.

Providing PC is recognised as: a core component of cancer care [11]: an essential service in the World Health Assembly cancer resolution [12]; and a core part of both Universal Health Coverage [13] and Primary Health Care [14]. It is also an important component of Sustainable Development Goal 3 on health [15], where investing in childhood cancer supports the attainment of multiple health-related targets, and identified as key service through the WHO Global Initiative for Childhood Cancer (GICC) [7]. The GICC recognises global inequalities and seeks to reach at least 60% survival probability at 5 years for children with cancer by 2030, while reducing suffering for all children with cancer [7]. The Cure*All* approach of the GICC calls for preparing health systems to respond to the needs of children along the entire pathway through co-ordinated multi-disciplinary care including early detection; diagnosis; treatment and PC; and survivorship [7].

The following principles of PPC apply to children with cancer: promotion of quality of life; provision of PC care across the continuum of care (from diagnosis through to bereavement); pain and symptom management; emotional support; social care; spiritual care; good communication with children and family; advance care planning; end-of-life care; and bereavement care. PPC should be provided across a range of care settings, wherever the child and their family need care, by an inter-disciplinary team providing support to the child, their families (including siblings) and significant others, and considers the financial impact of having a child with cancer. It should not be a last resort, but an essential component of care.

**Figure 1.**
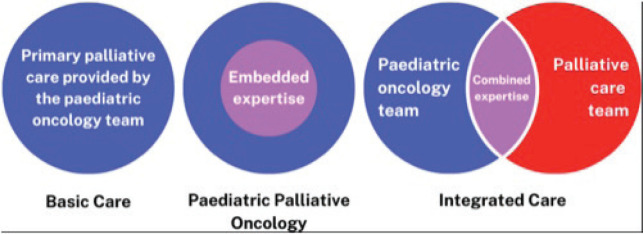
Possible models of PPC delivery in paediatric cancer care [16].

By providing PPC we aim to help children and their families achieve the best possible quality of life, however long that life might be; to relieve suffering, of body, mind and spirit, and care for them with competence and compassion. Supporting families throughout their journey is essential and early integration of PPC regardless of prognosis or treatment is considered best practice. This ideal timing of PPC integration is often challenged by cultural issues or a lack of resources [16]. Lacerda *et al* [16] discuss possible models of PPC delivery in children’s cancer including: 1) *Basic care*, where paediatric oncologists provide primary PC after basic education they may have received during their residency or fellowship 2) *Paediatric palliative oncology* where paediatric oncology clinicians have ‘embedded expertise’ gained from combined training in both oncology and PPC; 3) an *Integrated care model*, where oncology and PC teams work together for diagnosis, utilising the combined expertise to provide comprehensive care throughout the cancer disease trajectory (Figure 1).

## Challenges for the provision of PPC in cancer care

Access to PPC remains a significant challenge, both in HICs such as in the USA [17] and in LMICs [9, 18]. In the USA, challenges identified include the funding mechanisms, a lack of PPC programmes, difficulties in integrating PPC into existing paediatric oncology models and a lack of knowledge about PPC, discomfort at talking about death and cultural issues [17]. In LMICs there are a wide range of challenges that include a lack of:

- Policies on PPC;
- The recognition of the need for PPC and what it offers;
- Integration of PPC into the health system;
- Access to education;
- Accede to medicines, in particular to opioids; and
- Resources, such as financial [9].

Effective communication is critical in providing PPC [19]. However, many health professionals still find it challenging; finding the right words to explain life-threatening conditions or end-of-life options to children or their parents is tricky. If communication is poor, it may lead to impaired quality of life, inadequate management and mismanagement of the child’s and families care [19].

Delivering PPC can often give rise to ethical dilemmas [20]. Making decisions, such as when to stop treatment and discontinue invasive procedures can be challenging, especially when children may have views or desires that differ from their primary caregivers. Cultural beliefs also impact on acceptability of PC, along with accessing treatment and therefore late diagnosis.

PPC can be emotionally taxing and draining for healthcare providers as well who face daily suffering and death of their patients leading to distress and burnout [21]. PPC providers may also project their emotions into their private lives and relationships [22].

The WHO Conceptual Model for PC Development [23] identifies six key components to developing PC, including PPC, which can help to overcome these challenges: 1) Empower people and communities, 2) Health policies; 3) Research; 4) Essential medicines; 5) Education and training and 6) Integration of PPC into health services. This paper discusses examples of PPC provision in cancer care; research on PPC in cancer care; and examples of PPC education in cancer care.

## Examples of PPC provision in cancer care

Four examples of PPC are discussed from a low-income country (Uganda), two Middle-income countries from different regions (India and Lebanon) and a HIC (United States of America).

### Uganda

Few African countries have any form of PC programmes [18, 24]. In Uganda, the high prevalence of HIV/AIDS and cancer in the 1980s and early 1990s highlighted the need for PC. Thus, the first service – Hospice Africa Uganda started in 1993, establishing a systematic approach to PC in Uganda [25, 26], which became a model for the region.

In Uganda, PC for all hospitals was included in the National Health Sector Strategic Plan (HSSP) 2000/01–2004/05 [27, 28]. In 2004, the National Drug Policy and Authority Statute 1993 was amended so specially trained PC providers could prescribe morphine to improve access to opioids [28, 29]. The HSSP II, 2006–2011, expanded PC to all Hospitals and Health Centre IVs [26].

PPC in Uganda ranges from home-based care to hospital/facility-based care and integrated care. Several organisations provide PPC, e.g., The Uganda Cancer Institute, Hospice Africa Uganda, Mulago/Makerere PC Unit, Jinja Hospice, Kitovu Mobile Hospice, Joy Hospice, Kawempe Home Care and Mildmay Uganda, amongst others. Both Mildmay Uganda and Hospice Africa Uganda also train PPC providers at certificate, diploma and degree levels. It is important to note that Uganda has more than 50 tribes with distinct languages, cultures, norms and traditions, all influencing how they accept the concept of PPC [24]. Nevertheless, as the number of children with cancer rises, all stakeholders including government, civil society and child activists must take deliberate actions to increase access to appropriate PPC.

### Lebanon

PPC in Lebanon is emerging as an important approach to the treatment of childhood cancer, despite poor resources. There are a few major paediatric cancer centres in Lebanon with most located in urban areas, primarily in Lebanon’s Capital, Beirut, which offer childhood cancer treatment for Lebanon and surrounding regions using state-of-art cancer treatment within a multidisciplinary approach [30]. They provide disease-directed therapy, striving to integrate PPC within care through optimising symptom management, decision-making and advanced care-planning.

Given limited resources, PC services are limited to a few institutions and are often used in adult oncology settings typically at terminal stages. In one major paediatric cancer centre, an adult PC team collaborates to offer PPC services [30].

In the community, one non-governmental organisation provides home-based PC covering the capital with the possibility of telehealth for geographically far locations. However, demand exceeds bandwidth to optimise reach. This lack of accessibility along with cultural factors (such as the family involvement in the care during illness) accentuate parents’ role in delivering PPC, especially at home. A recent policy brief stipulates the provision of PC at patients’ residence as a ‘viable option’, valuing family ties [31]. Unpublished data on PPC tasks highlighted that parents in Lebanon engage in various PPC activities including managing treatment side effects, meeting emotional needs and praying with the child.

The Ministry of Public Health recently launched the national cancer plan with a chapter fully dedicated to PC, overarching both adults and paediatrics [32] which is a welcome development.

### India

According to a recent report on the National Cancer Registry Programme, childhood cancers account for 4.0% of all cancers in India [33]. Availability of PC becomes imperative given poor awareness, delayed diagnosis and treatment abandonment. However, less than 1% of those needing it, i.e., less than 42,500 children and their families have access to PC services in India.

Hyderabad runs one of the largest PPC programmes in the country through a well-integrated programme in a public cancer hospital, MNJ Institute of Oncology (MNJIO) and in Niloufer Children’s hospital, forming linkages in the community through a non-governmental organisation called ‘Pain Relief and PC Society’ (PRPCS). In MNJIO, every child admitted for cancer treatment is routinely screened for pain and offered PC support early in the course of illness. If the child relapses or has recurrent disease, there is a seamless transition to palliative and end-of-life care. The care continuum is ensured through a paediatric home-based programme and a ten bed children’s hospice – ‘Mandara’ (Hibiscus) – run by PRPCS. Many of these children come from remote districts of Telangana for treatment and are referred to Government-led district-based PC centres closest to home. Every service is provided with the help of a dedicated and trained multidisciplinary team and essential medications, such as oral morphine, are available even in most remote districts of the state. On average, 30 new children are referred for PC every month in MNJIO. Additionally, about 20 children are admitted to the hospice every month and on average 150 children are registered under the home care programme at any time point. The Hyderabad model of PPC is an example of providing comprehensive, cost effective and equitable coverage of care which benefits children and their families and is easily replicable.

### United States

Cancer remains the leading cause of death by disease among children in the United States, with approximately one in five children dying from their illness [34]. In the US, different entities including the American Society of Clinical Oncology, American Academy of Pediatrics, the Institute of Medicine, Association of Pediatric Hematology/Oncology Nurses and the National Consensus Project support the integration of PPC into health care. Specialist PPC is offered by PPC teams at 75% of childhood cancer centres [35]. Various models for the delivery of PC have been utilised and demonstrated to improve a variety of outcomes [36].

Different PPC programmes have varying inpatient, outpatient, community, virtual and home PPC services [35]. Multiple models of PC integration in oncology in the United States have been implemented, ranging from traditional consult-based models to oncologist-delivered primary PC, to referral for specialty PC in a consultative model, to systems-based trigger-prompted involvement, to embedded and fully integrated models of care [37]. Within the integrated model, care is led by the oncologist but they work closely with the PC team who attend weekly team meetings and visit the inpatient units on a daily basis. Such proximity is likely to help successful integration with education, relationship building and collaboration, as well as prompt identification of children who may benefit from PC [38]. A model combining this approach with systems-based triggers that prompts primary oncology teams to initiate PC consults is utilised at multiple US-based paediatric cancer centres. This model both combines the role of primary oncology teams and provides guidelines for when PC consultation may be beneficial.

Fully integrated, embedded or universal consultation models in paediatric oncology at a basic level with triggers for escalating levels of involvement, which effectively delivers individualised titrated PC services to patients is another approach. Thus, PC teams aim to reach the majority of high-risk patients early in their course of treatment but reserve full team resources for those who would benefit most. Combined training in paediatric haematology/oncology and hospice and palliative medicine (paediatric palliative oncology) can help facilitate this model [39]. A fully integrated model wherein PC consultation is embedded in teams such as the Haematopoietic Stem Cell Transplantation team and provides full consultative services to all patients is one that has great potential benefits but requires significant dedicated resources [40]. A recent report on fully integrated paediatric palliative oncology models includes embedding PPC in oncology in the following ways: (1) the *floating clinic* model where PPC experts float between inpatient, outpatient and home-based care, (2) a *disease-specific embedded PPC expert* where a PPC physician or nurse practitioner serves as a fully integrated member of the primary oncology team, (3) *trigger-based* or (4) *consultation-based* clinics where PPC consultations are triggered by pre-defined list of criteria or by oncology team and (5) *telehealth* PPC clinics [41].

## Examples of research in PPC provision in cancer care

There is a lack of robust evidence for PPC in cancer care [42]. Much of this evidence comes from high-income settings like UK, Europe, North America, Australia and New Zealand. Three examples of PPC research for children with cancer will be discussed from a range of settings: the ‘Assessing Doctors’ Attitudes on Palliative Treatment’ (ADAPT) study; review of literature on healthcare workers, parents and communities’ perspectives to PPC, and needs assessment and situational analysis for PPC in Ghana.

### ADAPT studies

Multiple barriers including lack of trained healthcare professionals (HCPs) and financial limitations exist in the integration of PPC. Additionally, underlying perceptions of PC and patient/parent refusal have also been identified as significant barriers to earlier integration of PC for children with cancer [43–47]. Historically, most research has been performed in high-income contexts, with little known about perceived barriers to PC for children in low- and middle-income contexts.

The *‘ADAPT’* survey was developed to assess physician perceptions of PC including physician comfort in providing PC, knowledge of PC principles and identification of perceived barriers to earlier integration [48–52]. The survey was originally developed for countries in Eastern Europe and Central Asia and has subsequently been translated and distributed in over 50 countries worldwide.

Some common global themes have emerged including a persistent misperception amongst physicians worldwide that PC is synonymous with end-of-life care [48, 50, 52]. Also there appears to be differences between what is viewed as ideal timing for PPC involvement and what happens in practice. Common barriers to integration exist including lack of home-based services, physician knowledge, access to PPC specialists, perceived family resistance and physician discomfort [49, 51, 52].

Identifying these barriers are important to provide interventions that can be similar worldwide, while also tailoring unique interventions, with the eventual goal of directing educational and advocacy efforts that are global in scope while also adaptable to specific regional/country level needs.

### Healthcare workers, parents and communities, perspectives about PPC

Existing literature regarding knowledge, attitudes and beliefs toward PPC amongst HCPs, parents and communities was recently summarised [53] to explore barriers challenging early PPC integration including lack of knowledge, negative attitudes and beliefs toward PPC. Sixty articles were included in the analysis, of which 82% (*n* = 49) were derived from HICs. Publications from LMICs were modest in number. Perspectives of HCPs were more extensively explored versus parents and community. The reports described confusion between PPC and end-of-life care. Factors associated with knowledge and attitudes included respondents’ demographic characteristics and patients’ clinical information. Although poor knowledge and negative attitudes toward PC were recurrent among HCP, patients, their parents and community, a positive attitude prevailed whenever respondents possessed accurate information about such care. Findings underscored a pressing need for prompt interventions among professionals and for timely awareness among non-professionals to alleviate children’s suffering.

### Needs assessment and situational analysis in Ghana

To understand the need for PPC in Ghana, a needs assessment and situational analysis was undertaken through World Child Cancer (WCC) by a collaborative team from International Children’s PC Network (ICPCN), WCC, the Ministry of Health, Ghana Health Service, Korle-Bu Teaching Hospital, Komfo Anokye Teaching Hospital and Greater Accra Teaching Hospital. PPC in Ghana is relatively new, with isolated and limited provision of services [54]. There are five trained paediatric oncologists in the country and access to healthcare services is limited, thus delaying care. Two focus group discussions with 21 participants and 17 in-depth interviews were conducted, with three overarching themes emerging: a conceptual model for PC, challenges to PPC and how to advance PPC in Ghana. Recommendations from this study are being reviewed, and a paper and policy brief being developed for PPC development in Ghana. Recommendations were aimed at the Ministry of Health and Ghana Health Services, for hospitals and service providers, education providers, researchers, donors and other stakeholders [55].

### Examples of education in PPC provision in cancer care

To provide PPC, we need essential resources and well-trained, competent personnel to provide such services. There are a wide range of education programmes available globally, including virtual, face-to-face and hybrid programmes, along with both generalist and specialist training. Three examples of education programmes spanning many countries are The Education in Palliative and End-of-Life Care (EPEC) – Paediatrics Programme; The ICPCN e-learning and webinars; and a 1-year Clinical Fellowship in Paediatric Palliative Medicine in South and South-East Asia*.*

### The EPEC – paediatrics programme

The EPEC-Paediatrics programme was funded through the US National Institutes of Health to support the delivery of primary PPC within the haematology/oncology setting. It was developed in response to a lack of advanced training in the prevention and treatment of pain and other distressing symptoms, along with the need for more widespread PC training for the paediatric haematology/oncology interdisciplinary team [56]. The curriculum is delivered through a combination of online and face-to-face sessions, and the training comprises of 24 core PC modules. EPEC-Paediatrics has been utilised as an end-user curriculum to improve HCPs knowledge of PPC in order that they can deliver primary PC. Additionally, the course is delivered in-person by ‘Master Facilitators’ at ‘Train-the-Trainer’ (TtT) conference sessions where EPEC-Paediatric training approach emphasises teaching participants advanced teaching skills such as how to utilise EPEC-Paediatrics to disseminate PPC concepts to others. The TtT Conferences focuses on delivering PPC material in engaging ways, fostering acquisition of attitudes, knowledge and skills (AKSs) that in turn help achieve desired clinical outcomes. Trainers (Paediatric clinicians) are provided with 22 adaptable PowerPoint presentations, all training videos and a Trainer’s handbook for each module to teach interdisciplinary teams across a range of settings.

EPEC-Paediatrics remains the largest and most comprehensive PPC training curriculum and dissemination project worldwide and has been translated in many languages. It has shown improvement in teaching ability and improved PPCAKSs in two core domains of PPC: communication and pain and symptom management. Most attendees of EPEC-Paediatric conferences report improvements in the clinical care of children with serious illnesses at their own institutions [57].

Additionally, EPEC-Paediatrics has been utilised as an integrated knowledge translation project where regional teams of 3–6 health professionals based at 15 paediatric oncology programmes in Canada became EPEC-Paediatrics Trainers and taught health curriculum to health professionals and implemented quality improvement (QI) projects [58]. The curriculum also demonstrated success in its regional adaptation, including the novel delivery of course content via a virtual bilingual format. The Eurasia-based course resulted in significant improvement in participant attitudes, knowledge of PPC and an understanding of ideal timing of PC consultation and comfort in providing PPC to children with cancer [59]. The EPEC – Paediatric curriculum is constantly being updated, adapted and expanded to encourage interdisciplinary global participation. 

### ICPCN’s e-learning and webinars

Education of HCPs lies at the heart of a coordinated response [23] to address PC needs of more than 21 million children and families globally [1]. The ICPCN’s education programme provides opportunities for both face-to-face and online training.

The ICPCN’s free e-learning platform, in its 12th year of existence, continues to grow with >9,000 participants from countries representing all 6 WHO geographic regions enrolled on the site. Courses cover several PPC topics, some available in 14 languages. Key to the advancement of the platform is focussing on optimising ‘user’ experience and engagement by adopting new technology and through the development of high-quality resources addressing existing gaps in CPC education.

The COVID-19 Pandemic necessitated a shift to virtual learning experiences with new initiatives being undertaken:

- The Global PC and COVID-19 series was developed in collaboration with ICPCN, the Worldwide Hospice PC Alliance, the International Association of Hospice and PC, and PC in Humanitarian Aid Situations and Emergencies. It included 124 experts from 27 countries who contributed to a series of webinars and accompanying briefing notes that provided globally relevant PC information and guidance within the context of the COVID-19 Pandemic.
- A set of monthly webinars linked to the book, CPC: an International case-based manual, edited by ICPCN [27] was developed in collaboration with contributors to the book. These webinars ran for 14 months and >2,800 attendees from >110 countries attended.
- Following the above webinars linked to the book, regular third Thursday monthly webinar meetings have continued with a range of topics identified and a diverse panel of speakers invited to share their expertise with a global audience.

Webinars are available to watch on ICPCN’s website and YouTube channel. Evaluation of ICPCN’s education programmes conducted in 2021 showed considerable change in knowledge, skills and attitudes with >80% saying it had improved their clinical practice. Importantly, online initiatives were seen as having a positive impact in terms of developing PPC service provision [60].

### 1-year clinical fellowship in paediatric palliative medicine for South and Southeast Asia

A 1-year fellowship programme was developed through an existing partnership between Two Worlds Cancer Collaboration, Canada and the Hyderabad Centre for PC, India, to train paediatricians as specialists and leaders in PPC in South and Southeast Asia. The format included an option of both a full time 1-year residential programme or a ‘Hybrid’ model.

The fellowship includes formal teaching, clinical rotations, mentorship, regular assessments of trainees and a scholarly project. Teaching includes 100 hours of weekly online classes, focusing on case-based learning and leadership skills. Mandatory 4 months of clinical rotations in PPC include 2 months in the regional center of PPC excellence in Hyderabad. A mentorship programme provides additional support, which continues beyond the fellowship through an early career mentorship group Trainees’ progression toward programme competencies is assessed through written and observed standardised clinical examinations. As part of research and QI training, fellows complete a scholarly project with support and supervision from experienced research mentors.

Since 2018, eight paediatricians (3 full time and 5 hybrid) have completed the fellowship, with three fellows currently in training. Graduated fellows have become regional and national leaders in PPC, developing new PPC programmes and implementing new PPC training in their home countries. The programme is endorsed by the Royal College of Paediatrics and Child Health (UK), which has strengthened the programme’s rigour and quality.

## Conclusion

Children with cancer and their families experience tremendous suffering, much of which can be relieved through the PPC. Providing PPC for paediatric cancer patients is, therefore, an ethical imperative. To address current gaps and unmet needs, we should look to successful clinical models of integration, continue to advance research and better understand the priorities of patients and families and continue to expand educational opportunities in this area. Together we can make a world of difference in the global integration of PPC into paediatric cancer care.

## Conflicts of interest

The author(s) declare that they have no conflict of interest.

## Funding

This work was not financed.
